# High Elastic Moduli of a 54Al_2_O_3_-46Ta_2_O_5_ Glass Fabricated via Containerless Processing

**DOI:** 10.1038/srep15233

**Published:** 2015-10-15

**Authors:** Gustavo A. Rosales-Sosa, Atsunobu Masuno, Yuji Higo, Hiroyuki Inoue, Yutaka Yanaba, Teruyasu Mizoguchi, Takumi Umada, Kohei Okamura, Katsuyoshi Kato, Yasuhiro Watanabe

**Affiliations:** 1 Institute of Industrial Science, The University of Tokyo, Tokyo, 153-8505, Japan; 2 Japan Synchrotron Radiation Research Institute, Hyogo, 679-5198, Japan

## Abstract

Glasses with high elastic moduli have been in demand for many years because the thickness of such glasses can be reduced while maintaining its strength. Moreover, thinner and lighter glasses are desired for the fabrication of windows in buildings and cars, cover glasses for smart-phones and substrates in Thin-Film Transistor (TFT) displays. In this work, we report a 54Al_2_O_3_-46Ta_2_O_5_ glass fabricated by aerodynamic levitation which possesses one of the highest elastic moduli and hardness for oxide glasses also displaying excellent optical properties. The glass was colorless and transparent in the visible region, and its refractive index *n*_*d*_ was as high as 1.94. The measured Young’s modulus and Vickers hardness were 158.3 GPa and 9.1 GPa, respectively, which are comparable to the previously reported highest values for oxide glasses. Analysis made using ^27^Al Magic Angle Spinning Nuclear Magnetic Resonance (MAS NMR) spectroscopy revealed the presence of a significantly large fraction of high-coordinated Al in addition to four-coordinated Al in the glass. The high elastic modulus and hardness are attributed to both the large cationic field strength of Ta^5+^ ions and the large dissociation energies per unit volume of Al_2_O_3_ and Ta_2_O_5_.

Glasses with high elastic moduli and high hardness values have been in demand for many years because the thickness of sheet glass with these properties can be decreased while maintaining its strength. Thinner and lighter glasses are desired for windows in buildings and cars, substrates for TFT displays, and covers of smart-phones^1^,^2^,^3^. The elastic modulus and hardness of a glass can be estimated with relatively good accuracy using semi-empirical models based on ionic-pair potentials that consider the chemical composition, density, and selected physical property data^4^,^5^,^6^. Particularly, the Young’s modulus *E* can be estimated using the Makishima and Mackenzie equation in which *E* is proportional to the atomic packing density and the sum of the partial dissociation energies of the components per unit volume^4^,^5^. The dissociation energies are in turn related to the bond strengths of the ionic pairs in the components. The Yamane and Mackenzie equation also indicates that the Vickers hardness (which is known to be directly related to the Young’s Modulus) is proportional to the square root of the bulk modulus *K*, shear modulus *G*, and the bond strengths of the components^7^. Therefore, in order to achieve high elastic moduli and high hardness values, the use of components with large dissociation energies and a high atomic packing density are key factors.

Alumina (Al_2_O_3_) has one of the highest dissociation energies among the oxides (*G*_*Al*2*O*3_ = 131 kJ/cm^3^)^4^. Accordingly, high elastic modulus and high hardness glasses generally include large quantities of Al_2_O_3_, as is found in *R*_2_O_3_-Al_2_O_3_-SiO_2_ glasses (*R* = rare earth ion, Y, or Sc)^8^,^9^,^10^,^11^. These glasses also have high atomic packing densities. However, because Al_2_O_3_ is considered an intermediate oxide according to Sun’s glass formation criteria, the glass forming ability of a composition typically decreases as the quantity of Al_2_O_3_ increases^12^. In addition, compositions with a large amount of Al_2_O_3_ are often difficult to melt due to their high melting temperatures. These issues have limited the fabrication of bulk glasses with high elastic moduli and high hardness values. Recent progress in containerless processing has, however, allowed the vitrification of low glass forming materials, including those without added network formers such as TiO_2_-based, Nb_2_O_5_-based, WO_3_-based, and Al_2_O_3_-based compositions, because heterogeneous nucleation from the melt can be avoided with this technique^13^,^14^,^15^,^16^,^17^,^18^. Thus, *R*_2_O_3_-Al_2_O_3_ glasses containing large quantities of Al_2_O_3_ have been prepared and found to exhibit superior mechanical properties as expected^19^,^20^. As a result, Al_2_O_3_-based glasses have attracted interest as high elastic moduli and high hardness materials. The properties of such glasses should be enhanced through the incorporation of additional components other than Al_2_O_3_ with high dissociation energies and high packing volumes. Herein, we describe the preparation of the new 54Al_2_O_3_-46Ta_2_O_5_ glass, which exhibits high elastic moduli and hardness values, using containerless processing. The thermal, optical, and mechanical properties of the glass are also reported. In addition, an approach to the design of glasses with higher elastic moduli and higher hardness is proposed on the basis of the results of the local structure analysis around aluminum performed using ^27^Al MAS NMR spectroscopy.

## Results

**Figure 1:**
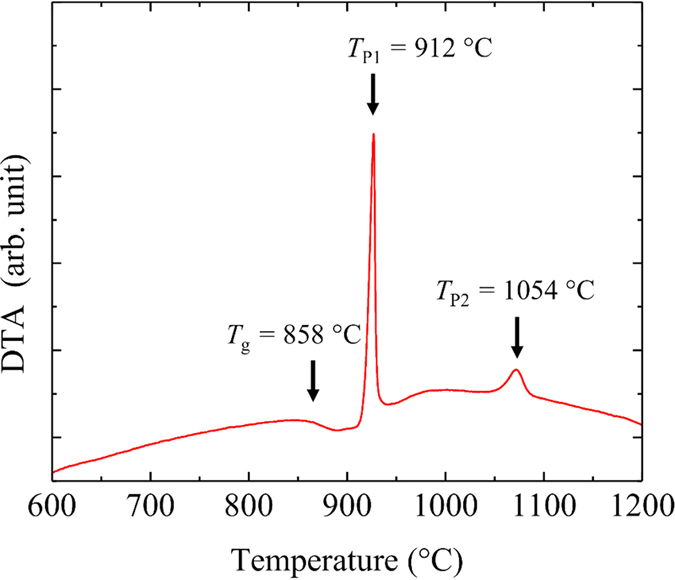
DTA curve for the 54Al_2_O_3_-46Ta_2_O_5_ glass showing *T*_*g*_, and the first (*T*_*P*1_) and second crystallization peaks (*T*_*P*2_).

**Figure 2:**
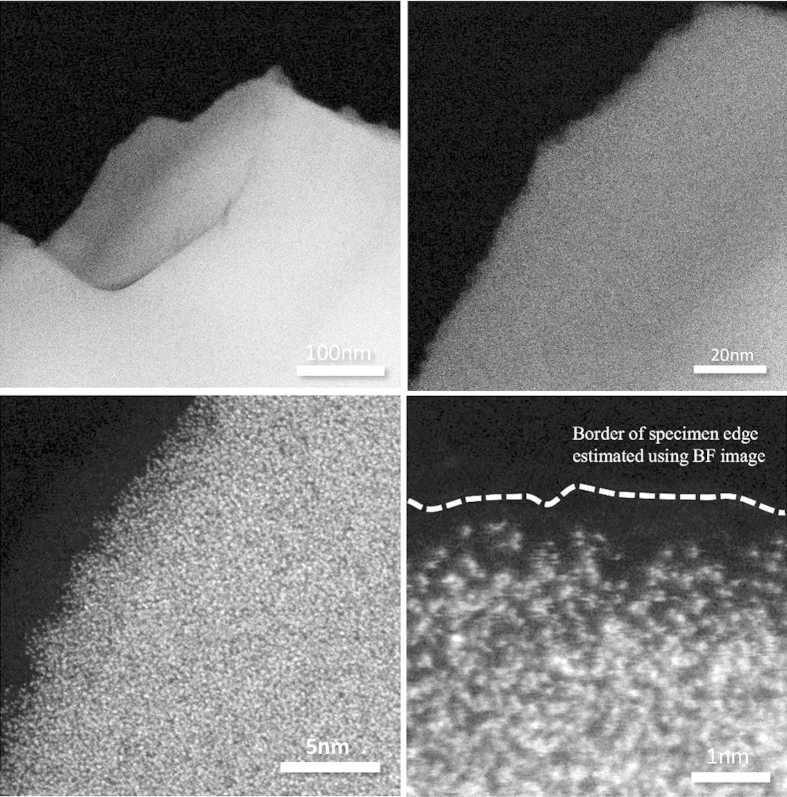
HAADF-STEM images at different magnifications for the 54Al_2_O_3_-46Ta_2_O_5_ glasses.

Figure 1 shows the Differential Thermal Analysis (DTA) curve for the 54Al_2_O_3_-46Ta_2_O_5_ glass. The glass transition temperature *T*_*g*_ is located at 858 °C, and the first *T*_*P*1_ and second *T*_*P*2_ crystallization peak are observed at 912 °C and 1054 °C, respectively. The difference between *T*_*P*1_ and *T*_*g*_ (Δ*T* = *T*_*P*1_ − *T*_*g*_) a measure of the thermal stability of the glass, is 54 °C, indicating the difficulty for vitrifying the glass using a conventional melting process. X-ray Diffraction (XRD) analysis confirmed that glass was totally amorphous and that the main phase of the crystallized sample after DTA was AlTaO_4_. The density of the annealed glass was *ρ* = 6.01 g/cm^3^. The composition of the glass samples measured by x-ray fluorescence (XRF) showed that the changes with respect to the nominal composition were less than 1 mol%. The microstructure of the fabricated glasses investigated through high-angle annular dark field scanning transmission electron microscopy (HAADF-STEM) is shown in Fig. 2. Observation through the HAADF-STEM has the advantage of achieving chemical contrast at the nanometric scale because it is very sensitive to the atomic number^21^. From the figure it can be observed that the glass is homogeneous at different scales and no phase-separation is observed. The randomly distributed bright points at the highest magnification are associated with the Ta atoms which have a much larger atomic number compared with the Al atoms (dark regions).

**Figure 3:**
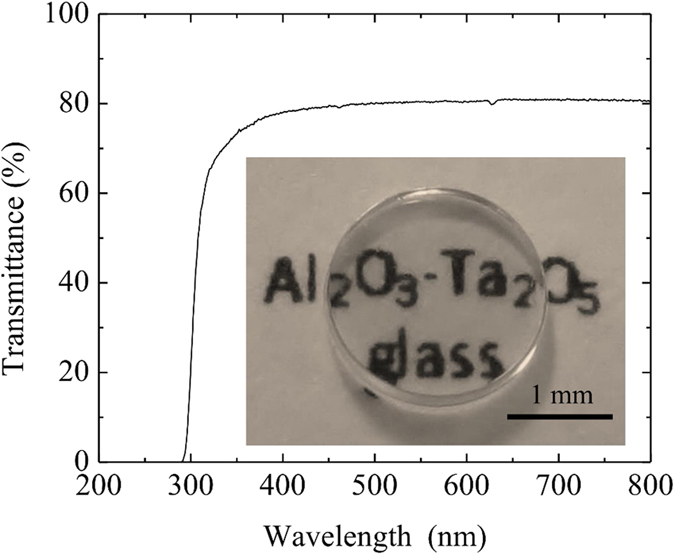
Transmittance spectrum of the 54Al_2_O_3_-46Ta_2_O_5_ glass in the UV/vis region. The inset picture shows the glass sample used for the transmittance experiment.

Figure 3 shows the transmittance spectrum of the 54Al_2_O_3_-46Ta_2_O_5_ glass in the ultraviolet-visible (UV/vis) region. The glass was transparent in the visible region and had a maximum apparent transmission of 81%. The maximum theoretical transmittance was also estimated to be 81% using the equation *R*_*max*_ = 1−[2*R*′/(1 + *R*′)], where *R*′ = [(*n*_*d*_ − 1)/(*n*_*d*_ + 1)]^2^, and the experimental refractive index *n*_*d*_ value of the glass which was found to be 1.94. The estimated value was similar as that of the experimental result, indicating that the apparent transmittance value was to the result of losses only due to sample surface reflection, and no light scattering occurred in the glass^22^. As observed in the inset of Fig. 3, the glass is colorless and transparent, which confirms that the valence state for all of the Ta ions is five, and no Ta^4+^ ions are present^23^. The optical bandgap energy was estimated to be 4.3 eV using the UV absorption edge located at 288 nm.

**Figure 4:**
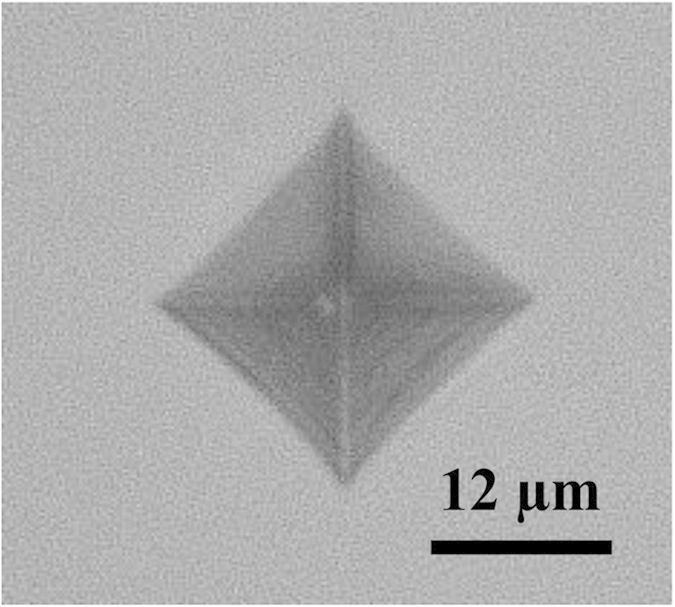
Vickers indentation imprint at 2.943 N for the 54Al_2_O_3_-46Ta_2_O_5_ glass.

The measured longitudinal velocity *V*_*P*_ and transversal velocity *V*_*S*_ of the 54Al_2_O_3_-46Ta_2_O_5_ glass were 5.86 km/s and 3.20 km/s, respectively. From these values and the experimental density, it was found that the Young’s modulus *E* was 158.3 GPa, the bulk modulus *K* was 124.1 GPa, the shear modulus *G* was 61.5 GPa, and the Poisson’s ratio *v* was 0.29. These values for the elastic moduli are considerably high and comparable to the maximum values in oxide glasses such as 40Y_2_O_3_-55Al_2_O_3_-5SiO_2_ and 28.5La_2_O_3_-71.5Al_2_O_3_, whose Young’s moduli were determined using Brillouin spectroscopy (169 GPa); however our measurement system showed that the Young’s modulus of those glasses were 145.5 GPa and 123 GPa respectively^1^,^9^,^10^. The Vickers hardness of the 54Al_2_O_3_-46Ta_2_O_5_ glass was 9.10 ± 0.05 GPa, which is also comparable to the maximum values reported for the oxide glasses; 81.8Al_2_O_3_-18.2Y_2_O_3_ (~9 GPa) and 29.3Al_2_O_3_-50.2SiO_2_-20.5Sc_2_O_3_ (9.4 GPa)^20^,^24^. Figure 4 shows indentation imprint for the 54Al_2_O_3_-46Ta_2_O_5_ glass at a load of 2.942 N. Extensive lines due to shear deformation on each face of the imprints are observed. In addition, at the same load, some of the imprints exhibited radial crack behavior^25^,^26^. No cracks were observed in any indentation below 1 N. The indentation cracking resistance (*CR*) was estimated to be 2.50 ± 0.13 N, which is comparable to a commercial Vycor glass^27^.

**Figure 5:**
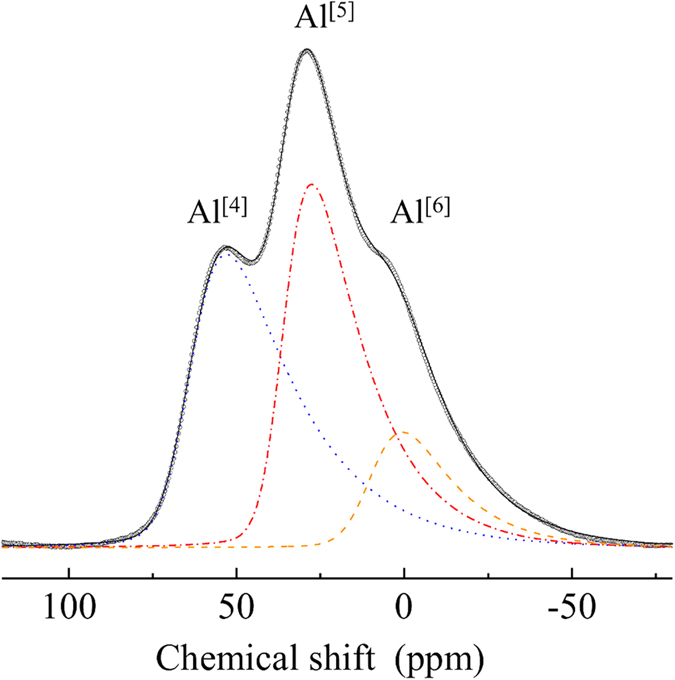
^27^Al MAS NMR spectrum of the 54Al_2_O_3_-46Ta_2_O_5_ glass. The total fitting curve corresponds to the sum of three Czjzek fitting curves: blue dots (Al^[4]^), red dashes and dots (Al^[5]^), and orange dashes (Al^[6]^).

The ^27^Al MAS NMR spectrum of the 54Al_2_O_3_-46Ta_2_O_5_ glass is presented in Fig. 5. Although the spectrum is broad due to the amorphous nature of the glass, two distinctive peaks and a small shoulder were observed. These peaks and the shoulder were assigned to 4-coordinated Al (Al^[4]^), 5-coordinated Al (Al^[5]^), and 6-coordinated (Al^[6]^), respectively^28^,^29^,^30^. The spectrum was decomposed into the three components using the “dmfit” program applying a simple Czjzek model^31^,^32^. The thin dotted lines in the spectrum correspond to each of the components. The fitting, values for *δ*_*iso*_ (isotropic chemical shift), dCSA (width of the Gaussian distribution of *δ*_*iso*_), and *v*Q* (quadrupolar product in kHz) were determined to be 64.8 ppm, 15 ppm, and 1134 kHz for Al^[4]^; 36.7 ppm, 12 ppm, and 985 kHz for Al^[5]^; and 10.3 ppm, 15 ppm, and 973 kHz for Al^[6]^, respectively^33^. Based on the integration of the peak areas, the fractions of Al^[4]^, Al^[5]^, and Al^[6]^ were estimated to be 44.1%, 41.9%, and 14.0%, respectively. The estimated average oxygen coordination number for Al was 4.7. The fractions of Al^[5]^ and Al^[6]^ were considerably larger than those observed in other aluminate glasses; Al typically forms AlO_4_ tetrahedra in *M*O-Al_2_O_3_ (*M* = Ca, Sr and Ba) glasses^30^. While Al^[5]^ and Al^[6]^ have been observed in some Al_2_O_3_-containing glasses, such as *R*_2_O_3_-Al_2_O_3_ (*R* is a rare earth ion or Y), *R*_2_O_3_-Al_2_O_3_-SiO_2_, and CaO-Al_2_O_3_-SiO_2_, the fraction of Al^[5]^ has generally ranged from 3 to 30%, and that of Al^[6]^ from 1 to 2%^24^,^34^,^35^. The structure of the 54Al_2_O_3_-46Ta_2_O_5_ glass may therefore be due to not only the presence of AlO_4_ networks but also result in part from the high oxygen coordination of Al. The mechanism of glass formation with retention of large fractions of Al^[5]^ and Al^[6]^ is interesting and thus will be the subject of further investigations.

## Discussion

These combined results indicate that the 54Al_2_O_3_-46Ta_2_O_5_ glass have good mechanical properties, high transparency and a high refractive, with an unconventional amount of Al^[5]^ and Al^[6]^ species. In order to understand the origin of the good mechanical properties of the glass the results are analyzed within the context of the Makishima and Mackenzie model *e.g.* the atomic packing density and dissociation energy per unit volume of the glass components.

The atomic packing density *C*_*g*_ was calculated from the density using the formula *C*_*g*_ = *ρ*(Σ*x*_*i*_*V*_*i*_)/*M*, where *M* is the molecular weight of the glass and *x*_*i*_ is the molar fraction of the component *i*. The ionic volume *V*_*i*_ of an oxide is *N*_*A*_ (4/3) 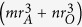, where *N*_*A*_ is Avogadro’s number, *m* and *n* are the number of atoms in the *A*_*m*_*O*_*n*_ oxide, *r*_*A*_ is the ionic radius of the cation, and *r*_*O*_ is the ionic radius of oxygen. Shannon and Prewitt ionic radii were used^36^. The coordination numbers for Ta and O in the 54Al_2_O_3_-46Ta_2_O_5_ glass were assumed to be 6 and 2, respectively, and the fractions of the coordination numbers for Al estimated from the results of the ^27^Al MAS NMR were used. The atomic packing density *C*_*g*_ was found to be 0.586, which is significantly larger than those for conventional oxide glasses (i.e., for SiO_2_ glass, *C*_*g*_ is 0.452). The small molar volume of Ta_2_O_5_ and the large fraction of highly coordinated Al are thought to contribute to the high packing density of the 54Al_2_O_3_-46Ta_2_O_5_ glass. It has been suggested that the formation of highly coordinated Al in aluminate glasses is promoted by the large cationic field strength, as observed in *R*_2_O_3_-Al_2_O_3_-SiO_2_ glasses^8^,^9^,^10^,^11^. Ta^5+^ also has large cationic field strength because of its small ionic radius and high valence state. Accordingly, Ta_2_O_5_ likely contributes to the high packing density of the 54Al_2_O_3_-46Ta_2_O_5_ glass via the formation of a large number of highly coordinated Al atoms.

A high content of Ta_2_O_5_ is also characteristic of the 54Al_2_O_3_-46Ta_2_O_5_ glass. The dissociation energy of Ta_2_O_5_ is substantially large (95.6 kJ/cm^3^)^11^. The elastic moduli of the glass were estimated using the Makishima and Mackenzie equation given by *E* = 2*C*_*g*_(Σ*x*_*i*_*G*_*i*_). Here *G*_*i*_ is the dissociation energy of each component oxide. Values of 131 kJ/cm^3^, 125 kJ/cm^3^ and 119.2 kJ/cm^3^ were used for *G*_*Al*2*O*3_ with Al in coordination of 4, 5, and 6 respectively^11^,^37^,^38^. The calculated Young’s modulus *E* of the 54Al_2_O_3_-46Ta_2_O_5_ glass was 131.9 GPa, which was approximately 17% less than the experimentally determined value, but still in relatively good agreement. A more accurate model may be necessary for estimation of the atomic packing density that includes the real contribution of the more highly coordinated cations. The energy contribution ratios of Al_2_O_3_ and Ta_2_O_5_ to the Young’s modulus were also estimated using the Makishima and Mackenzie equation and found to be 62% and 38%, respectively. It should be noted that the contribution of Al_2_O_2_ is not that high, while that of Ta_2_O_5_ is considerably high, which is unlike most other binary aluminate glasses with high elastic moduli. For example, a 28.5La_2_O_3_-71.5Al_2_O_3_ glass, which has one of the highest reported Young’s modulus values among the oxide glasses, has the following contribution: 16.71% from La_2_O_3_ and 83.3% from Al_2_O_3_. It has been previously accepted that a large contribution by Al_2_O_3_ is necessary to achieve a high elastic modulus for binary aluminate glasses, such as *R*_2_O_3_−Al_2_O_3_. However, a simple estimation of the energy contribution of the components in 54Al_2_O_3_-46Ta_2_O_5_ glass revealed that an appropriate component, like Ta_2_O_5_, can increase the elastic modulus even if the dissociation energy contribution of Al_2_O_3_ is small.

In summary, a glass with composition 54Al_2_O_3_-46Ta_2_O_5_ was fabricated using an aerodynamic levitation technique. Its glass transition temperature *T*_*g*_ was 858 °C, and crystallization occurred at 54 °C above *T*_*g*_, indicating a low glass forming ability. The glass is colorless and highly transparent in the visible region and has a refractive index *n*_*d*_ of 1.94. The Young’s modulus *E*, bulk modulus *K*, shear modulus *G*, and Poisson’s ratio *v* of the 54Al_2_O_3_-46Ta_2_O_5_ glass were determined via ultrasonic pulse-echo overlap analysis and were found to be 158.3 GPa, 124.1 GPa, 61.5 GPa, and 0.29, respectively, while the Vickers hardness of the glass was found to be 9.1 GPa. These elastic moduli and Vickers hardness values are quite high and comparable to the maximum values of conventional oxide glasses. In addition, an indentation cracking resistance of 2.5 N was estimated from the indentation experiments. Furthermore, ^27^Al MAS NMR spectroscopic analysis revealed that the fractions of Al^[4]^, Al^[5]^, and Al^[6]^ in the 54Al_2_O_3_-46Ta_2_O_5_ glass were 44.1%, 41.9%, and 14.0%, respectively, and the average oxygen coordination number of the Al cations was 4.7. Notably, the fractions of Al^[5]^ and Al^[6]^ are considerably large compared to those observed in conventional oxide glasses, and may form because of the large cationic field strength of Ta^5+^. These results indicated that Ta_2_O_5_ was a key contributor to the high elastic moduli and high hardness values of the glass because the addition of Ta_2_O_5_ increases the packing density via formation of Al atoms that are highly coordinated with oxygen and because the Ta_2_O_5_ itself has a large dissociation energy. Moreover, a simple estimation of the energy contributions of the components in the 54Al_2_O_5_-46Ta_2_O_5_ glass using the Makishima and Mackenzie equation also suggested that the use of appropriate components can increase the elastic moduli even if the contribution of Al_2_O_3_ is small. These results provide insight into the design and fabrication of harder glasses based on both the local structure and the dissociation energies of the components.

## Methods

### Glass synthesis

Glasses were fabricated using an aerodynamic levitation furnace described elsewhere^23^. High-purity (99.99%) *α*-Al_2_O_3_ and Ta_2_O_5_ powders were mixed stoichiometrically with the chemical composition 54Al_2_O_3_-46Ta_2_O_5_, pelletized using a hydrostatic press, and annealed at 1050 °C for 12 h in air. Pieces obtained from the crushed pellets were levitated in an oxygen gas flow and melted using two CO_2_ lasers at approximately 2000 °C. The melt was rapidly solidified by shutting off the lasers at a cooling rate of approximately 300 °C/s in order to obtain fully vitrified samples. The obtained spherical glasses (2 mm in diameter) were colorless and transparent. Glass formation was confirmed via Cu *Kα* XRD analysis (Rigaku, RINT 2000). In order to rule out any compositional changes of the glass during the melting process, X-ray fluorescence experiments (JEOL, JSX-3100RII) were performed on glass samples under vacuum conditions. Glasses with composition 40Y_2_O_3_-55Al_2_O_3_-5SiO_2_, 28.5La_2_O_3_-71.5Al_2_O_3_, and 29.3Al_2_O_3_-50.2SiO_2_-20.5Sc_2_O_3_ were also fabricated using the levitation technique for comparative purposes.

### Scanning transmission electron microscopy observation

In order to verify the homogeneity of the fabricated glasses observation with a scanning transmission electron microscope (JEOL, ARM-200CF) coupled with a high-angle annular dark field (HAADF) detector was performed. The microscope was equiped with a spherical aberration corrector (Ceos, Gmbh) and a cold field emission gun was used. The probe-forming aperture angle was 24.5 mrad while the HAADF and bright field (BF) detectors spanned through 68–280 and 0–17 mrad respectively. The spatial resolution of the present observation was approximately 0.1 nm. Glass powders were dropped into a perforated amorphous carbon films supported in Cu grids. No sputtering or heating was applied to the samples prior to the observation.

### Thermal and physical properties

The glass transition temperature *T*_*g*_ and crystallization temperature *T*_*p*_ were determined via DTA at a heating rate of 10 °C/s (SII, TG6300). Prior to the analysis of the physical and structural properties, the glasses were annealed at 10 °C above the *T*_*g*_ in order to relax the stress introduced during quenching. The density *ρ* was determined using gas pycnometry (Micrometrics, AccuPyc II 1340). The experimental error associated with the density measurements was smaller than 0.01 g/cm^3^. The experimental densities for the 40Y_2_O_3_-55Al_2_O_3_-5SiO_2_, 28.5La_2_O_3_-71.5Al_2_O_3_ and 29.3Al_2_O_3_-50.2SiO_2_-20.5Sc_2_O_3_ glasses were 4.95 g/cm^3^, 4.22 g/cm^3^ and 3.04 g/cm^3^ respectively. The transmittance spectrum of an approximately 300 *μ*m-thick sample was obtained in the range from 200 nm to 800 nm using a UV/vis spectrometer (Shimadzu, UV3100PC). The refractive index dispersion was determined via spectroscopic ellipsometry (J. A. Woolam, M-2000U).

### Elastic moduli measurement

The pulse-echo overlap technique was used to obtain the ultrasonic velocities of the glass^39^. A 50 *μ*m-thick ultrasonic transducer (LiNbO_3_ 10 °Y-cut) and a 300 *μ*m-thick glass were pasted at opposite corners of an edge truncated tungsten carbide (WC) block using a conductive epoxy resin. The ultrasonic echoes of the longitudinal *P* and shear *S* waves from the transducer were reflected by the glass and observed using a digital oscilloscope. The longitudinal velocity *V*_*P*_ and transversal velocity *V*_*S*_ were determined by dividing the thickness of the samples by the observed travel time of the waves. The longitudinal modulus *L* (*C*_11_) and shear modulus *G* (*C*_44_) were estimated using the equations 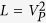 and 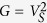. The Young’s modulus *E*, bulk modulus *K*, and Poisson’s ratio *v* were calculated using the equations *E* = *G*(3*L* − 4*G*)/(*L* − *G*), *K* = *L* − (4/3)*G*, and *v* = (*L* − 2*G*)/(*L* − *G*), respectively. The obtained Young’s modulus *E* for the 40Y_2_O_3_-55Al_2_O_3_-5SiO_2_, 28.5La_2_O_3_-71.5Al_2_O_3_ and 29.3Al_2_O_3_-50.2SiO_2_-20.5Sc_2_O_3_ glasses were 145.6 GPa, 123 GPa and 133.2 GPa respectively.

### Indentation behavior

Indentation experiments were performed on a Shimazu DUH HMV-1 Vickers tester at 23 °C and approximately 60% relative humidity. Optical-grade polished samples with a thickness of approximately 500 *μ*m were used. The dwell time was set at 15 s. The Vickers hardness values *H*_*V*_ were calculated from the diagonal lengths of the imprints at a load of 0.980 N. At least 20 indents were made for measuring *H*_*V*_. The indentation cracking resistance *CR* values were estimated from cracking probability curves using the method proposed by Wada *et al.*^40^. Here, *CR* is defined as the load required to generate two radial cracks on average or to achieve a 50% cracking probability. Each data point on the cracking probability curves developed in the present study also represents 20 indentation imprints. The reported value of *CR* was obtained by averaging the *CR* values determined from sigmoidal fittings of the cracking probability curves for three different samples. The imprints were observed by optical microscopy.

### Al local structure

^27^Al MAS NMR spectroscopy of the glass was performed on a JEOL JNM-ECA 500 spectrometer equipped with MAS probe head at 11.74 T (500 MHz). The spinning rate was 15 kHz, and a 4-mm-diameter zirconia rotor was used. The NMR spectra were recorded using *π*/6 pulses (0.4 *μ*s) and a relaxation delay of 1 s, and 4000–12000 signals were accumulated. The 27Al chemical shift *δ*_*iso*_ in parts per million (ppm) was referenced to an external 1 M AlCl_3_ solution (−0.1 ppm).

## Additional Information

**How to cite this article**: Rosales-Sosa, G. A. *et al.* High Elastic Moduli of a 54Al_2_O_3_-46Ta_2_O_5_ Glass Fabricated via Containerless Processing. *Sci. Rep.* **5**, 15233; doi: 10.1038/srep15233 (2015).
